# Human Mast Cell Line HMC1 Expresses Functional Mas-Related G-Protein Coupled Receptor 2

**DOI:** 10.3389/fimmu.2021.625284

**Published:** 2021-03-15

**Authors:** Maud A. W. Hermans, Astrid C. van Stigt, Sanne van de Meerendonk, Benjamin Schrijver, Paul L. A. van Daele, Petrus M. van Hagen, Marloes van Splunter, Willem A. Dik

**Affiliations:** ^1^ Section of Allergy & Immunology, Department of Internal Medicine, Erasmus MC, Rotterdam, Netherlands; ^2^ Laboratory of Medical Immunology, Department of Immunology, Erasmus MC, Rotterdam, Netherlands

**Keywords:** mast cell (MC), MRGPRX2, compound 48/80, qwf, HMC1, LAD2, HuMC, latrunculin

## Abstract

The Mas-related G-protein-coupled receptor X2 (MRGPRX2) is prominently expressed by mast cells and induces degranulation upon binding by different ligands. Its activation has been linked to various mast cell-related diseases, such as chronic spontaneous urticaria, atopic dermatitis and asthma. Therefore, inhibition of MRGPRX2 activity represents a therapeutic target for these conditions. However, the exact pathophysiology of this receptor is still unknown. In vitro research with mast cells is often hampered by the technical limitations of available cell lines. The human mast cell types LAD2 and HuMC (human mast cells cultured from CD34+ progenitor cells) most closely resemble mature human mast cells, yet have a very slow growth rate. A fast proliferating alternative is the human mast cell line HMC1, but they are considered unsuitable for degranulation assays due to their immature phenotype. Moreover, the expression and functionality of MRGPRX2 on HMC1 is controversial. Here, we describe the MRGPRX2 expression and functionality in HMC1 cells, and compare these with LAD2 and HuMC. We also propose a model to render HMC1 suitable for degranulation assays by pre-incubating them with latrunculin-B (Lat-B). Expression of MRGPRX2 by HMC1 was proven by RQ-PCR and flowcytometry, although at lower levels compared with LAD2 and HuMC. Pre-incubation of HMC1 cells with Lat-B significantly increased the overall degranulation capacity, without significantly changing their MRGPRX2 expression, phenotype or morphology. The MRGPRX2 specific compound 48/80 (C48/80) effectively induced degranulation of HMC1 as measured by CD63 membrane expression and β-hexosaminidase release, albeit in lower levels than for LAD2 or HuMC. HMC1, LAD2 and HuMC each had different degranulation kinetics upon stimulation with C48/80. Incubation with the MRGPRX2 specific inhibitor QWF inhibited C48/80-induced degranulation, confirming the functionality of MRGPRX2 on HMC1. In conclusion, HMC1 cells have lower levels of MRGPRX2 expression than LAD2 or HuMC, but are attractive for *in vitro* research because of their high growth rate and stable phenotype. HMC1 can be used to study MRGPRX2-mediated degranulation after pre-incubation with Lat-B, which provides the opportunity to explore MPRGRX2 biology in mast cells in a feasible way.

## Introduction

Mast cells are innate-type leukocytes that reside at barrier surfaces of the body, mainly the skin and mucosa. Here, they contribute to local immune responses induced by exogenous or physical triggers that disturb local tissue homeostasis (1). Mature mast cells harbor a wide array of surface membrane receptors that enable them to respond to many different triggers, provoking their effector functions (1). Depending on the type of stimulus, different types of activation can be induced, typically resulting in degranulation whereby different kinds of preformed mediators can be rapidly expelled (1, 2). Histamine, leukotrienes, prostaglandins and other vasoactive substances that are released by mast cells contribute to symptoms of anaphylaxis (3). Next to the rapid degranulation of preformed molecules, mast cells can initiate a slower pro-inflammatory response. This involves synthesis and secretion of cytokines and chemokines that subsequently activate neighboring cells and recruit and activate infiltrating immune cells (1). Furthermore, mast cells strongly interact with fibroblasts in wound healing responses, and mast cell-derived proteases are important in eradicating toxic venoms (4, 5). All the functional characteristics above illustrate the importance of mast cells in the control of variety of physiological and pathophysiological effects.

In modern medicine, mast cells have a mainly negative image due to their role in allergic disease. Consequently, the IgE-mediated route of mast cell activation has gained the most scientific attention in the past decades (6). More recently, it has become clear that other routes of mast cell activation can also lead to degranulation and, thereby, anaphylaxis. An intriguing G-protein coupled receptor termed the Mas-Related G-protein coupled Receptor X2 (MRGPRX2) was initially considered to be expressed exclusively on mast cells in humans (7). More recently, functional MRGPRX2 membrane surface expression has also been described on eosinophils and basophils, although no quantitative comparison was made with mast cells (8). It has also become clear that the expression and functionality of MRGPRX2 on human MC extracted from different bodily tissues can vary: skin mast cells appear to have the highest MRGPRX2 expression whereas lung mast cells do not express MRGPRX2 (9). MRGPRX2 has many ligands, including hormones and neuropeptides, small molecule drugs and venoms (7). It has extensively been proven that binding of one of these ligands to MRGPRX2 induces mast cell degranulation, but little to none cytokine production by mast cells (10, 11). However, the intracellular signaling cascade downstream of the receptor has not yet been fully elucidated.

Mast cell activation through MRGPRX2 presumably plays a major role in pseudo-allergic reactions induced by small molecule drugs, including neuromuscular blocking agents and insect venom (10, 12). However, it is unknown why not all individuals experience such hypersensitivity reactions upon administration of the mentioned MRGPRX2 ligands and, more importantly, how to prevent them. Next to hypersensitivity reactions to exogenous substances, MRGPRX2 activation has been linked to chronic spontaneous urticaria (13, 14), atopic dermatitis (15) and allergic asthma (16). Thereby, it forms a potentially interesting target for treatment of various mast cell related diseases. In order to develop effective therapies, the biological behavior of the receptor and its downstream intracellular mechanisms need to be elucidated. This requires profound insight into MRGPRX2 biology in mast cells, which could ideally be obtained from comprehensive *in vitro* approaches, where a standardized experimental setting is provided.

*In vitro* research with human mast cells is severely hampered by the fact that they typically display low proliferative activity. Moreover, the delicate nature of these cells is easily disturbed by physical triggers, including mechanical stress (17). Many researchers culture human mast cells (HuMC) from CD34^+^ myeloid progenitor cells derived from buffy coats, cord blood or bone marrow to study human mast cell biology *in vitro* (18). These HuMC, which develop from progenitors cells by use of specific culture medium conditions, indeed resemble normal human mast cells closely and have been shown to express MRGPRX2 accordingly (19). Unfortunately, the method to differentiate them is time consuming and expensive. Furthermore, there is appears to be a limited time span in which they can be optimally used for experiments, e.g. at the age of 12-16 weeks (20).

To avoid these practical difficulties, a few human mast cell lines are available. Laboratory of Allergic Diseases type 2 cells (LAD2) were derived from CD34+ cells isolated from bone marrow aspirate of a patient with aggressive systemic mastocytosis without any detectable KIT mutation (21). LAD2 cells stably express IgE receptor type 1 (FcεR1), display a granular appearance, require stem cell factor (SCF) for survival and proliferate slowly (doubling time of 2 weeks). Furthermore, LAD2 cells have been proven to functionally express MRGPRX2 (22, 23). As such, the LAD2 cell line could be considered as reasonably resembling normal human mast cells. However, a main disadvantage for the use of LAD2 for *in vitro* research is their very slow proliferation rate, making massive expansion troublesome (21).

Another human mast cell line is Human Mast Cell 1 line (HMC1), that was derived from a patient with mast cell leukemia. HMC1 cells harbor either 1 or 2 activating mutations in KIT (dependent on the exact subtype of HMC1 line), rendering them independent of SCF for survival and making them highly proliferative with a doubling time of 2-3 days (24, 25). HMC1 displays a dedifferentiated phenotype as evidenced by marginal FcεR1 expression and a spindle-shaped hypogranular appearance. While they display only little release of histamine, tryptase and other typical mast cell mediators upon degranulation, HMC1 cells can produce large amounts of cytokines upon activation (24, 26). Although it is an immature, immortalized human mast cell line with consequential dissimilarities as compared with normal human mast cells, HMC1 could represent an attractive model for MRGPRX2 research, mainly due to its high *in vitro* proliferation rate. To our knowledge, only one other group has previously investigated the expression of MRGPRX2 in HMC1. In their paper, they briefly described the functionality of HMC1 as nonexistent, although based on rather limited data (27).

It is thus clear that there is a need for a feasible *in vitro* mast cell model to study MRGPRX2 biology in this cell type. Although HMC1 have practical advantages over LAD2 and HuMC when used for *in vitro* studies, it should first be ensured whether they express and respond to MRGPRX2 and how this relates to LAD2 and HuMC. The goal of this study was to examine the expression and functionality of MRGPRX2 on HMC1, and compare this to LAD2 and HuMC. Moreover, we developed a method to improve HMC1 degranulation capacity which renders them a potentially useful model to functionally study MRGPRX2.

## Methods

### Cell Culture

HMC1.2 cells [kindly provided by Dr. Butterfield, Mayo Clinic Rochester, Minnesota (25)], were cultured in Roswell Park Memorial Institute medium (RPMI, Lonza, Verviers, Belgium) supplemented with 4-(2-Hydroxyethyl)piperazine-1-ethanesulfonic acid (HEPES, Lonza, Verviers, Belgium), 10% inactivated fetal calf serum (FCS), 50 μM β‐mercapto‐ethanol (Sigma‐Aldrich, St. Louis, Missouri), and 1% antibiotics (penicillin/streptomycin, Lonza, Verviers, Belgium). We have used HMC1.2 cells, which harbor the G560V and D816V mutations in KIT. For the rest of this manuscript, the term ‘HMC1’ will be used. HMC1 cells were passaged 2-3 times a week and maintained at a density of 1-1.2*10^6 cells/ml.

LAD2 cells [kindly provided by Drs. Kirshenbaum and Metcalfe, National Institute of Allergy and Infectious Diseases, Bethesda, Maryland (21)] were cultured in Stem Pro‐34 medium, supplemented with 2.6% nutrient supplement (both Life Technologies, Grand Island, New York), 2 mmol/L ultraglutamine (Lonza, Verviers, Belgium), recombinant human stem cell factor (rhSCF) (100 ng/ml, R&D systems, Abbingdon, UK), and 1% antibiotics (penicillin/streptomycin). Cells were passaged once a week, preserving 50% old medium and adding 50% new medium with freshly added rhSCF (100 ng/ml).

Primary human mast cells (HuMC) were established from CD34^+^ myeloid progenitors cells isolated from buffy coats of peripheral blood obtained from healthy blood bank donors, based on the protocol published by Folkerts et al. (28). First, peripheral blood mononuclear cells (PBMCs) were isolated from the buffy coats by ficoll density separation. Subsequently, lineage depletion (CD3/CD19/CD14) was conducted by magnetic-activated cell sorting with magnetic beads (Human Lineage Cell Depletion Kit; Miltenyi Biotec, Galdbach, Germany). The negative fraction, considered to be enriched for CD34^+^ hematopoietic stem cells, was exposed to selection pressure using specific culturing conditions, enabling the progenitor cells to develop into HuMC. During the first month, cells were cultured in culture medium I, containing StemSpan medium (StemCell technologies, Vancouver, British Columbia, Canada) supplemented with 1% penicillin/streptomycin, recombinant human interleukin 6 (rhIL-6, 50 ng/mL, Peprotech, Rocky Hill, NJ), rhIL-3 (10 ng/mL, Peprotech), and rhSCF (100 ng/mL, Peprotech). Cells were kept at a density of 0.5-1*10^6^ cells/ml. With every passage, 50% of the old medium was preserved and 50% of new medium with freshly included rhSCF, rhIL-6 and rhIL-3 was added. Hereafter, culture medium I was gradually substituted by culture medium II by again adding 50% new medium to 50% preserved medium with every passage. Culture medium II contained Iscove’s modified Dulbeccos medium with GlutaMAX-I (IMDM, Lonza, Verviers, Belgium), 50 μM β‐mercapto-ethanol, 0.5% FCS, 1% Insulin-Transferrin-Selenium (Life Technologies, Carlsbad, CA), with 1% antibiotics (penicillin/streptomycin), with fresh addition of 100 ng/ml rhSCF and 50 ng/ml rhIL-6. HuMC were confirmed to have a specific mast cell phenotype after ~12 weeks in culture, by toluidine blue staining and flowcytometric analysis of FcεR1, KIT and MRGPRX2 expression (see below). HuMC aged 12-16 weeks were used for further experiments.

### Receptor Profiling

Surface membrane expression of KIT (CD117), FcεRI and MRGPRX2 was assessed by flowcytometry on LAD2, HMC1, HuMC and PBMCs (as triple negative control). Hereto, 2*10^5^ cells were suspended in 500 μl of phosphate-buffered saline (PBS), followed by centrifugation at 200g (HuMC), 300g (HMC1 and LAD2) or 500g (PBMC) for 5 minutes. After removal of the supernatant, cell pellets were resuspended in 90 μl PBS. Subsequently, cells were stained by adding 5 μl monoclonal mouse-anti-human MRGPRX2-alexafluor488 antibody (R&D systems, Mineapolis U.S.), 1 μl monoclonal mouse-anti-human CD117-Pe-Cy7 antibody (Beckman Coulter, Brea, U.S.) and 2 μl monoclonal mouse-anti-human FcεRI-APC-A antibody (Biolegend, San Diego, U.S), followed by 15 minutes incubation at room temperature (RT) in the dark. Thereafter, cells were washed with 2 mL PBS, resuspended in 150 μl FACS flow buffer (1% FCS, 0.09% NaN_3_, PBS) and analyzed on a flowcytometer (LSRII, Becton Dickinson, Franklin Lakes, New Jersey, U.S). The mean fluorescent intensity (MFI) for each antibody was determined by measuring at least 2*10^4^ single live cells. Per cell type, unstained and unstimulated stained conditions were always included. For each time point, the experiment was conducted two fold.

### Toluidine Blue Staining

Firstly, cells were washed and resuspended in PBS in a concentration of 2*10^5^ cells/ml. In order to make cytospin slides, 50 µl of cell suspension was loaded into loading chambers, followed by centrifugation for 5 minutes at 14g using a cytofuge (Nordic Immunological laboratories, the Netherlands), thus transferring cells onto glass slides. Next, the cells were fixed with Mota’s fixative for 10 minutes, which was then removed *via* indirect rinsing with deionized water. Subsequently, 2-3 droplets of toluidine blue dye were added onto the cells for 20 minutes, followed by indirect rinsing with deionized water. Cells were tapped dry and a second coverslip was placed over the stained cells, mounted with warmed gelatin (Boom, Meppel, Netherlands). Stained slides were stored at 4 °C. The images were analyzed using an Axiovert microscope with AxioCAM MR5 (Zeiss, Oberkochen, Germany) and photographed at 100x magnification, within five days after the staining procedure.

### HMC1 Degranulation Assay Using Latrunculin-B

HMC1 are recognized to display poor degranulation activity (26). To optimize HMC1 degranulation, we explored the effect of the zinc-finger toxic protein Latrunculin B (Lat-B) on degranulation induced by calcium ionophore A23187 (1μM, Sigma Aldrich, Missouri, U.S), since this has proven to be a potent general mast cell activator in our hands (29). Lat-B is a macrolide-type protein derived from the Red Sea sponge *Latrunculina magnifica* that influences actin in the cytoskeleton and thereby theoretically enhances degranulation capacity (30). Degranulation was determined by β-hexosaminidase release and CD63 expression, as described below. Degranulation assays were performed two to six fold for each time point.

### MRGPRX2-Dependent Mast Cell Stimulation

Stimulation experiments were performed using the MRGPRX2 specific ligand compound 48/80 (C48/80, Sigma Aldrich, Missouri, U.S). For LAD2, a concentration range of 0.1-1.5-10 μg/ml C48/80 was used, while for HMC1 1-5-10-50-100 μg/ml of C48/80 was used. Based on the results found with LAD2 and HMC1, a concentration range of 0.1-1-10-50-100 μg/ml C48/80 was used to stimulate HuMC. As a positive control, cells were stimulated with A23187 (1 μM). Stimulation time ranged from 15 and 30 to 60 minutes and all stimulations were performed at 37°C. Due to a limited amount of cells, caused by the aforementioned slow proliferation rates of LAD2 and HuMC, not all concentrations and stimulation time-points could be tested for all three different types of mast cells.

To further prove the functionality and specificity of MRGPRX2 activation, additional inhibition experiments were conducted by pre-incubation with the MRGPRX2 specific antagonist QWF (Boc-Gln-D-Trp(Formyl)-Phe benzyl ester trifluoroacetate salt, Santa Cruz biotechnology, Texas, U.S (10, 31)) for 10 minutes, at concentrations of 10, 25 and 100 μM. An unstimulated condition containing an equal amount of the QWF diluent, dimethyl sulfoxide (DMSO), was included in every experiment as a control condition, not exceeding the maximal concentration of 0.28% DMSO during stimulation of the cells. Degranulation was assessed by β-hexosaminidase release and CD63 expression, as described below.

### β-Hexosaminidase Release Assay

Mast cell degranulation was measured by β-hexosaminidase assay, essentially as previously described (29). The cells were diluted in PBS containing 1.5 μM Ca^2+^ and seeded in a 96-well plate at a concentration of 2*10^4^ cells/well. C48/80 or A23187, also diluted in PBS, were added in the indicated concentrations. After stimulation, the plate was centrifuged for 5 minutes at 300g. Hereafter, 50 μl of supernatant was transferred to another 96-wells plate (Nunc MaxiSorp™ flat-bottom) containing 50 μl 4 μM p‐nitrophenyl N‐acetyl‐ β‐D‐glucosamine (p‐NAG, Sigma Aldrich, Missouri, U.S) in citrate buffer (pH 4.5). The cell pellet was lysed using 150 μl 0.1% Triton X solution (Sigma Aldrich, Missouri, U.S). Hereafter, 50 μl of cell lysate was transferred to another 96-wells plate containing 50 μl 4 μM pNAG solution. After incubation for 90 minutes at 37°C, 100 μl of glycine 400 mM was added to each well to end the reaction. Optical density (OD) values were measured at 405 and 620 nm, using ELISA plate reader (VersaMax microplate reader, Molecular Devices, San Jose, U.S.). The relative β‐hexosaminidase release was calculated as follows:

$$\%\;\beta -\text{hexosaminidase release}=\frac{2x\;\Delta \text{supernatan}(=\text{OD supernatant}-\text{OD blank condition})}{\left(\Delta \text{supernatant}+(4*\;\Delta \text{cell lysate}\right)}$$

### Surface Membrane CD63 Expression

Upregulation of CD63 expression was used as a measure for mast cell degranulation, as described previously (32). In brief, the cells were suspended in PBS with 1.5 μM Ca^2+^ to a concentration of 2x10^4^ cells in 500 μL per tube. The cells were stimulated as described above. Thereafter, the cells were fixed with 1% paraformaldehyde (PFA, Sigma Aldrich, Missouri, U.S), washed with FACS buffer and stained with 2 μl monoclonal Mouse-anti-Human CD63-APC antibody (ThermoFisher, Waltham, Massachusetts) and with 1 μl monoclonal mouse-anti-human CD117-PE-Cy7 as a positive control. Surface membrane expression of CD63 was measured using a flowcytometer (Canto II, Becton Dickinson, Franklin Lakes, New Jersey).

Because the percentage of CD63 positive cells on unstimulated mast cells could vary, especially for HMC1, a combination of CD63 expression plus increase in sideward scatter was used to determine the percentage of degranulated mast cells, and the relative increase of CD63 expression was calculated. **Figure S1** shows a representative example of flowcytometry results.

The x-fold increase of CD63 expression was calculated as follows:

$$\text{x}-\text{fold increase}=\frac{\text{percentage CD}63\;\text{positive cells of total stimulated condition}}{\text{percentage CD}63\;\text{positive cells of total unstimulated condition}}$$

### Statistical Analysis

Statistical analysis was performed in GraphPad Prism version 5 or IBM SPSS statistics version 25. Per statistical analysis, data were checked for having a Gaussian distribution using a Shapiro-Wilk test in combination with a Q-Q plot. If data were normally distributed, a paired t-test was performed for the comparison of two groups, and a two-way ANOVA with Bonferroni post-hoc test for the comparison of multiple groups. When data had a non-Gaussian distribution, Mann Whitney U tests were used to statistically compare continuous non-paired variables, and the Wilcoxon signed rank test for paired variables.

## Results

### Expression of the Mast Cell Specific Receptors MRGPRX2, FcϵRI and KIT

**Figure 1:**
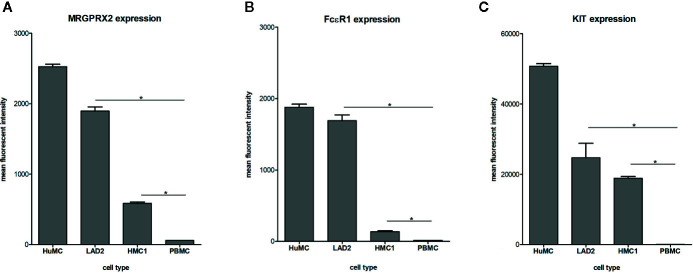
Expression of MRGPRX2 **(A)** FcϵR1 **(B)** and KIT **(C)** by different mast cell lines: HuMC, LAD2, HMC1. Receptor expression was assessed by triple staining and measured by flowcytometry. The mean fluorescent intensity (MFI) with SEM is shown of single, live population of HuMC (n=3) and LAD2, HMC1 and PBMCs (all n=6), stained with MRGPRX2-AF488, FcϵRI-APC and cKIT-PE-Cy7. *p<0.05 for MFI compared with PBMC.

Surface membrane expression of MRGPRX2, FcϵRI and KIT receptor was detected on all three different types of mast cells, albeit at different levels, with HuMC showing the highest and HMC1 showing the lowest expression level (**Figure 1**). All three receptors were hardly expressed by PBMC, confirming its use as negative control. Since MRGPRX2 surface expression by HMC1 was relatively low, we conducted real-time quantitative polymerase chain reaction (RQ-PCR) to confirm its expression. MRGPRX2 mRNA was expressed by HMC1 as well as HuMC (serving as positive control), while no MRGPRX2 mRNA was detected in PBMC (**Figure S2**).

### Optimized HMC1 Degranulation Assay

**Figure 2:**
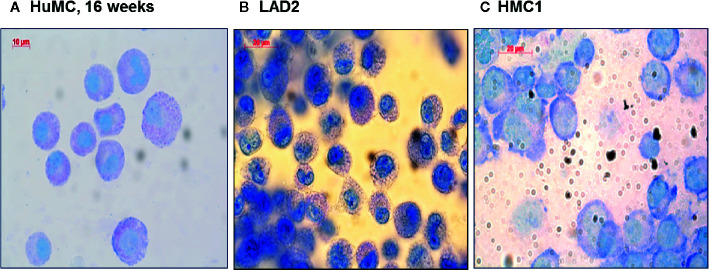
Morphologic appearance of different mast cell lines: HuMC, LAD2, HMC1. Toluidine blue staining of HuMC **(A)** and LAD2 **(B)** show a typical granular pattern. HMC1 **(C)** shows a different morphology as compared to HuMC and LAD2, in particular, granules are absent.

**Figure 3:**
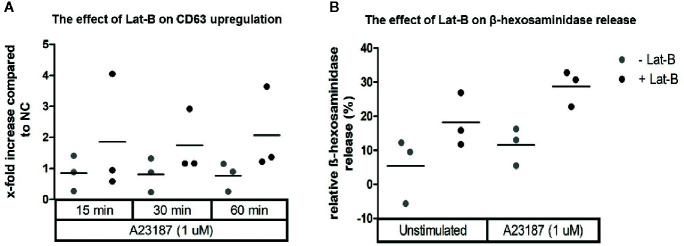
Calcium ionophore A23187 induced degranulation of HMC1 with or without Lat-B. HMC1s pre-incubated with 2 µg/ml Lat-B for 24 hours showed an overall higher fold induction of CD63 expression upon stimulation with 1 μM A23187, as compared to HMC1 cells without pre-incubation **(A)**. The same effect was seen on β-hexosaminidase release **(B)**. No substantial differences were seen regarding the length of stimulation time.

Since HMC1 generally have a poor degranulation capacity, we first aimed at optimizing the degranulation assay for this cell type, in order to subsequently examine MRGPRX2 functionality. HMC1 cells displayed little granularity in comparison to LAD2 and HuMC (**Figure 2**), which might explain their limited immediate degranulation capacity (26). Pre-incubation of HMC1 cells with 2 µg/ml Lat-B for 24 hours approximately doubled the stimulatory effect of A23187 on degranulation, as shown by enhanced CD63 surface membrane expression as well as β-hexosaminidase release (**Figures 3A, B**).

**Figure 4:**
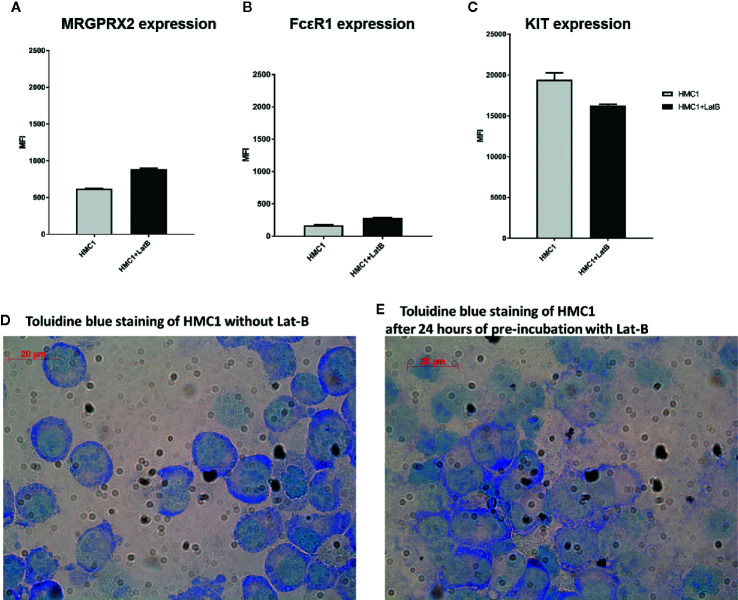
Latrunculin-B does not induce phenotypical changes to HMC1 cells. The receptor expression of HMC1s with and without pre-incubation with 2 µg/ml Lat-B for 24 hours is shown (n=3, Mean Fluorescent Intensity with SEM). The expression of MRGRPX2 **(A)** and FcϵRI **(B)** is slightly upregulated after pre-incubation with Lat-B, but not statistically significant. The expression of KIT is decreased after pre-incubation with Lat-B, but again not statistically significant **(C)**. The morphological appearance of HMC1s is not changed upon pre-incubation with Lat-B **(D, E)**.

Lat-B pre-incubation resulted in a marginal increase in MRGPRX2 and FcϵR1 expression and a slight decrease in KIT expression on HMC1, but none of these changes were statistically significant (**Figures 4A–C**). Toluidine blue assessment revealed no gross morphological alterations of HMC1 upon incubation with Lat-B (**Figures 4D, E**).

### MRGPRX2 Functionality

**Figure 5:**
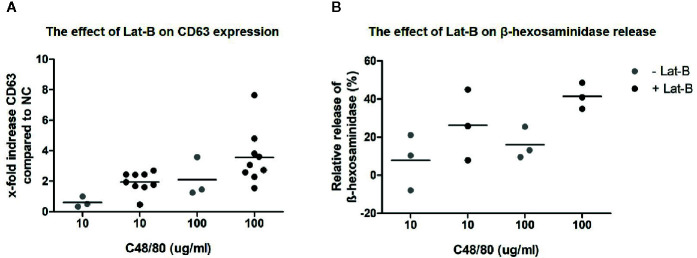
C48/80 induced degranulation of HMC1 with and without Lat-B. **(A)** Stronger upregulation of CD63 in response to C48/80 stimulation was seen after pre-incubation with Lat-B, with a dose dependent effect. **(B)** The relative β-hexosaminidase release was increased after pre-incubation with Lat-B compared to without Lat-B, but not reaching statistical significance.

After establishing that incubation with Lat-B provided a suitable model for HMC1 degranulation, the potential of the MRGPRX2 specific agonist C48/80 to induce degranulation of HMC1 with and without Lat-B was investigated. C48/80 did induce surface membrane CD63 expression as well as β-hexosaminidase release by HMC1, which was enhanced by Lat-B pre-incubation (**Figures 5A, B**).

**Figure 6:**
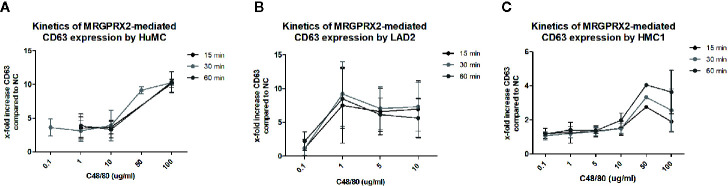
Different kinetics of MRGPRX2 mediated degranulation of three mast cell lines: HMC1, LAD2, HuMC. The mean x-fold increase of CD63 expression upon stimulation with C48/80 is shown for all three cell lines at different concentrations of C48/80 and different time periods of stimulation. The error bars depict SEM. n=2 for HuMC and LAD2s and n=3 for HMC1s. **(A)** HuMCs of two donors were used. Both showed a similar pattern, with significant CD63 upregulation only for the highest concentrations of C48/80. There was no difference for different periods of stimulation. **(B)** LAD2s responded to much lower concentrations of C48/80, and 30 minutes proved the optimal period of stimulation. However, considerable variation was seen between the experiments. **(C)** HMC1s, pre-incubated with Lat-B, only showed CD63 upregulation upon stimulation with C48/80 at a concentration of 50 or 100 µg/ml for 60 minutes.

To further explore MRGPRX2 functionality, combined titration and time course experiments with C48/80 and the MRGPRX2 specific antagonist QWF were conducted. These experiments revealed that 100 times lower concentration of C48/80 was required to induce degranulation of LAD2, compared with HMC1; 1 μg/mL versus 100 μg/mL, respectively (**Figure 6**). Furthermore, C48/80-induced degranulation appeared optimal after 15-30 minutes of stimulation for LAD2, whereas at least 60 minutes stimulation was required for HMC1 (**Figure 6**). Unexpectedly, HuMC required higher concentrations of C48/80 than we anticipated on the basis of the previously observed MRGPRX2 expression (**Figure 1**), while the duration of stimulation did not influence the level of CD63 expression. Overall, HuMC and LAD2 reached much higher levels of CD63 surface membrane expression than HMC1.

**Figure 7:**
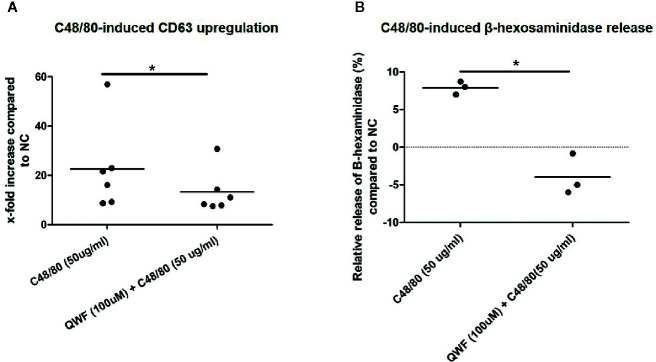
QWF effectively inhibits C48/80 induced degranulation. **(A)** QWF effectively and significantly inhibits C48/80 induced degranulation of HMC1s, as measured by CD63 expression (*p 0.03, Wilcoxon rank test). **(B)** QWF effectively and significantly inhibits C48/80 induced degranulation of HMC1s, as measured by β-hexosaminidase release (*p 0.01). Relative increases compared with the negative control are shown, the dashed line thus represents the negative control condition.

To confirm the specificity of C48/80 for MRGPRX2, inhibition experiments were performed using the MRGPRX2-specific antagonist QWF to pre-incubate the cells. Indeed, C48/80-induced degranulation of HMC1 was significantly inhibited by QWF, as measured by CD63 surface membrane expression (**Figure 7A**) as well as β-hexosaminidase release (**Figure 7B**). The inhibitory effect of QWF displayed a dose-dependent manner (**Figure S3**). To check for nonspecific stimulation of β-hexosaminidase release or CD63 expression by the diluent DMSO or the inhibitor QWF, compound control experiments were performed with both substances in the highest concentrations used in this study. It was noted that only QWF appeared to increase CD63 surface membrane expression in itself (**Figure S4A**), whereas neither DMSO nor QWF induced any relevant β-hexosaminidase release (**Figure S4B**). To prevent bias by this nonspecific effect of QWF on CD63 expression, the concentration of QWF that did not induce more CD63 expression than the negative controls was selected as the maximal concentration used in inhibition experiments. However, this probably explains the fact that the effect of QWF on C48/80-induced CD63 expression was smaller than on β-hexosaminidase release as can be appreciated from **Figure 7**, although the biological explanation for this finding is lacking.

## Discussion

Here, for the first time to our knowledge, we have demonstrated that MRGPRX2 is functionally expressed by the HMC1 cell line. Since MRGPRX2 activation mainly induces mast cell degranulation and little to none cytokine production (11), a model was sought to enhance the degranulation by HMC1. Although these cells displayed diminished degranulation capacity compared with LAD2 and HuMC, we found HMC1 suitable for degranulation studies after pre-incubation with Lat-B.

The main advantage of HMC1 over other mast cell lines, is the fact that they have a 10-fold higher division rate than LAD2 and HuMC. Unfortunately, working with HMC1 also has several caveats, most importantly its poor degranulation capacity. Furthermore, its high constitutive KIT activity might have a negative influence on MGRPRX2-mediated activation (33). Until now, it was unclear whether HMC1 expressed MRGPRX2. In fact, one published study briefly described MRGPRX2 functionality on HMC1 as nonexistent, although this appeared to be based on somewhat unusual techniques and stimulation compounds for the investigation of MRGPRX2 functionality in human mast cells (cortistatin and bovine adrenal medulla docosapeptide) (27). However, some degree of MRGPRX2 expression could be demonstrated by realtime-PCR in this study, although the researchers did not use other techniques such as flowcytometry to confirm their findings (27).

Overall, HuMC theoretically form a more representative cell type to investigate MRGPRX2 biology *in vitro*. However, HuMC required much higher concentrations of C48/80 than we expected from the high levels of MRGPRX2 expression that were initially found. It must be noted that the HuMC which were used for the initial measurement of receptor expression (**Figures 1** and **S2**) were derived from different donors than the HuMC which were used for the stimulation assays (**Figure 6**). Upon determining the MRGPRX2 expression on the latter HuMC batches, we found a considerably lower MRGPRX2 expression than on the HuMC from the first two donors, using flowcytometry as well as RQ-PCR. The MRGPRX2 expression of unstimulated HuMC could vary up to a factor 10 between different donors, whereas the KIT expression was comparable across all donors (data not shown). Whether this can be influenced by tweaking the culture media or that it is merely an inter-individual difference needs to be further investigated. This inter-donor variability of MRGPRX2 expression shows that although resembling mature human mast cells, HuMC derived from buffy coats are an unpredictable system for studying MRGPRX2-mediated degranulation, potentially causing incompatible results of assays.

To avoid differences in MRGPRX2 expression throughout experimental settings, the use of a phenotypically stable cell line such as HMC1 is more attractive, especially knowing that the degranulation capacity can be optimized with Lat-B. The effect of Lat-B on cell degranulation has been described before, and is presumed to be caused by its binding to monomeric actin, inhibiting F-actin polymerization and thereby disrupting micro-filament mediated processes (30). Disruption of this F-actin barrier is necessary to allow exocytosis of granules. F-actin disruption, followed by rapid cortical actin disassembly, is therefore an important feature of mast cell and basophil degranulation, as has been demonstrated for example by IgE-mediated degranulation in rat basophilic leukemia cell line (RBL) cells (34). Accordingly, Frigeri and Apgar showed augmentation of IgE-mediated degranulation by RBL cells after pre-incubation with Lat-B, with an increase in β-hexosaminidase release from 20% to 40% (35). Next to this, Smrz et al. found that SCF could solely induce degranulation of HuMC after incubation with Lat-B (36). This will probably not be of significance to HMC1, since they are independent of SCF due to the activating mutations in KIT which are the hallmark of this cell line.

Although we have attempted to exclude functional effects of Lat-B on mast cell behavior, it remains an artificial method which is different from the *in vivo* situation in various ways. Furthermore, the degranulation of HMC1 will never be an optimal representation of wild-type cell lines. For example, several endogenous and exogenous stimuli can influence MRGPRX2 activity in the human body, such as other cytokines and hormones (15). This might be different for HMC1 due to their immature phenotype and continuous KIT activity. Moreover, the kinetics of HMC1 degranulation are different compared with other mast cell types as was demonstrated here. Gaudenzio et al. previously demonstrated that the degranulation kinetics of HuMC can also vary according to different stimuli as well (11).

Dependent on the aim of a study and the stimuli used, researchers will have to choose the most suitable cell line based on the characteristics of each cell type. HuMC theoretically form the most optimally representative cells for biological research, but since this is not a stable cell line, the phenotype and receptor expression can vary between donors. Furthermore, it is yet unknown to what extent the receptor expression fluctuates according to the maturation of the HuMC. LAD2 represents a more stable cell line, but is less useful for *in vitro* research due to its low proliferation rate.

In conclusion, HMC1 functionally expresses MRGPRX2, albeit at a lower level than LAD2 and HuMC. The use of HMC1 has the benefits of a phenotypically stable cell line with high proliferative activity, making them feasible to conduct extensive *in vitro* experiments. Incubation with the actin-disruptive macrolide Lat-B enhances the degranulation capacity of HMC1 without functionally changing their phenotype. Although HMC1 have different degranulation kinetics compared with wild-type mast cells, they form a feasible model for the investigation of mast cell degranulation and MRGPRX2 biology.

## Author’s Note

All authors are members of the Academic Centre of Excellence for Allergic Diseases.

## Data Availability Statement

The original contributions presented in the study are included in the article/**Supplementary Material**. Further inquiries can be directed to the corresponding author.

## Author Contributions

MH, AS and WD created the concept of this study. AS, SM, BS and MS performed the experiments and created the figures. The research was discussed on multiple occasions with the whole team of authors throughout the study, adjusting the experiments where necessary. MH created the first draft of the manuscript. AS, SM, BS, PD, PH, MS and WD critically revised the manuscript. All authors contributed to the article and approved the submitted version.

## Conflict of Interest

The authors declare that the research was conducted in the absence of any commercial or financial relationships that could be construed as a potential conflict of interest.

## Supplementary Material

Supplementary Figure 1 Representative example of flowcytometry results for CD63 upregulation upon C48/80 induced degranulation of HMC. Degranulated mast cells are in Q2: they are CD63 positive, and have increase sideward scatter. **(A)**. For the negative control condition, only 0.290% of all cells are degranulated. **(B)**. After stimulation with C48/80, 25.7% of HMC1 are in Q2, thus considered degranulated.

Supplementary Figure 2 Confirmation of MRGPRX2 RNA expression by HMC1. A clear mRNA expression of MRGPRX2 was shown to be present in HMC1 (n=3), but not in PBC (n=2), which were used as negative control. Mean with SEM is shown for CT values.

Supplementary Figure 3 QWF dose-dependently inhibits C48/80 induced HMC1 degranulation. A titration of QWF doses was performed to identify the optimally inhibiting dose of QWF. Two representative experiments are shown. HMC1s, pre-incubated with Lat-B, were first incubated with QWF for 10 minutes and subsequently stimulated with C48/80 for 60 minutes. A dose-dependent decrease of QWF on CD63 expression was found.

Supplementary Figure 4 Control conditions of the compounds used in this study. The effect of DMSO and QWF alone on the activation of HMC1 was investigated as an extra control. The highest concentrations used in the study were taken: 0.28% DMSO, and 100 µg/ml QWF. **(A)** Both DMSO and QWF did not induce relevant β-hexosaminidase release (n=3). **(B)** QWF appeared to induce a nonspecific CD63 upregulation although not statistically significant compared with the diluent control DMSO (n=6).

## References

[1] Huber M Cato ACB Ainooson GK Freichel M Tsvilovskyy V Jessberger R. Regulation of the pleiotropic effects of tissue resident mast cells. J Allergy Clin Immunol (2019) 144(4S):S31–S45. 10.1016/j.jaci.2019.02.004 30772496

[2] Moon TC Befus AD Kulka M. Mast cell mediators: their differential release and the secretory pathways involved. Front Immunol (2014) 5:569. 10.3389/fimmu.2014.00569 25452755 PMC4231949

[3] Metcalfe DD Peavy RD Gilfillan AM. Mechanisms of mast cell signaling in anaphylaxis. J Allergy Clin Immunol (2009) 124(4):639–46; quiz 47-8. 10.1016/j.jaci.2009.08.035 PMC2788154 19815110

[4] Komi DEA Khomtchouk K Santa Maria PL. A Review of the Contribution of Mast Cells in Wound Healing: Involved Molecular and Cellular Mechanisms. Clin Rev Allergy Immunol (2020) 58(3):298–312. 10.1007/s12016-019-08729-w 30729428

[5] Metz M Piliponsky AM Chen CC Lammel V Abrink M Pejler G. Mast cells can enhance resistance to snake and honeybee venoms. Science (2006) 313(5786):526–30. 10.1126/science.1128877 16873664

[6] Caslin HL Kiwanuka KN Haque TT Taruselli MT MacKnight HP Paranjape A. Controlling Mast Cell Activation and Homeostasis: Work Influenced by Bill Paul That Continues Today. Front Immunol (2018) 9:868. 10.3389/fimmu.2018.00868 29755466 PMC5932183

[7] McNeil BD Pundir P Meeker S Han L Undem BJ Kulka M. Identification of a mast-cell-specific receptor crucial for pseudo-allergic drug reactions. Nature (2015) 519(7542):237–41. 10.1038/nature14022 PMC4359082 25517090

[8] Wedi B Gehring M Kapp A. The pseudoallergen receptor MRGPRX2 on peripheral blood basophils and eosinophils: Expression and function. Allergy (2020) 75(9):2229–42. 10.1111/all.14213 32003863

[9] Varricchi G Pecoraro A Loffredo S Poto R Rivellese F Genovese A. Heterogeneity of Human Mast Cells With Respect to MRGPRX2 Receptor Expression and Function. Front Cell Neurosci (2019) 13:299. 10.3389/fncel.2019.00299 31333418 PMC6616107

[10] Porebski G Kwiecien K Pawica M Kwitniewski M. Mas-Related G Protein-Coupled Receptor-X2 (MRGPRX2) in Drug Hypersensitivity Reactions. Front Immunol (2018) 9:3027. 10.3389/fimmu.2018.03027 30619367 PMC6306423

[11] Gaudenzio N Sibilano R Marichal T Starkl P Reber LL Cenac N. Different activation signals induce distinct mast cell degranulation strategies. J Clin Invest (2016) 126(10):3981–98. 10.1172/JCI85538 PMC5096814 27643442

[12] Che D Rui L Cao J Wang J Zhang Y Ding Y. Cisatracurium induces mast cell activation and pseudo-allergic reactions via MRGPRX2. Int Immunopharmacol (2018) 62:244–50. 10.1016/j.intimp.2018.07.020 30032049

[13] Vena GA Cassano N Di Leo E Calogiuri GF Nettis E. Focus on the role of substance P in chronic urticaria. Clin Mol Allergy (2018) 16:24. 10.1186/s12948-018-0101-z 30473632 PMC6240950

[14] Fujisawa D Kashiwakura J Kita H Kikukawa Y Fujitani Y Sasaki-Sakamoto T. Expression of Mas-related gene X2 on mast cells is upregulated in the skin of patients with severe chronic urticaria. J Allergy Clin Immunol (2014) 134(3):622–33.e9. 10.1016/j.jaci.2014.05.004 24954276

[15] Wang Z Babina M. MRGPRX2 signals its importance in cutaneous mast cell biology: Does MRGPRX2 connect mast cells and atopic dermatitis? Exp Dermatol (2020) 29(11):1104–11. 10.1111/exd.14182 32866307

[16] An J Lee JH Won HK Kang Y Song WJ Kwon HS. Clinical significance of serum MRGPRX2 as a new biomarker in allergic asthma. Allergy (2020) 75(4):959–62. 10.1111/all.14084 31605391

[17] Fowlkes V Wilson CG Carver W Goldsmith EC. Mechanical loading promotes mast cell degranulation via RGD-integrin dependent pathways. J Biomech (2013) 46(4):788–95. 10.1016/j.jbiomech.2012.11.014 PMC3646572 23261248

[18] Yin Y Bai Y Olivera A Desai A Metcalfe DD. An optimized protocol for the generation and functional analysis of human mast cells from CD34(+) enriched cell populations. J Immunol Methods (2017) 448:105–11. 10.1016/j.jim.2017.06.003 PMC5654741 28629733

[19] Elst J Sabato V Faber MA Bridts CH Mertens C Van Houdt M. MRGPRX2 and Immediate Drug Hypersensitivity: Insights from Cultured Human Mast Cells. J Investig Allergol Clin Immunol (2020) 0. 10.18176/jiaci.0557 32732181

[20] Schmetzer O Valentin P Smorodchenko A Domenis R Gri G Siebenhaar F. A novel method to generate and culture human mast cells: Peripheral CD34+ stem cell-derived mast cells (PSCMCs). J Immunol Methods (2014) 413:62–8. 10.1016/j.jim.2014.07.003 25038510

[21] Kirshenbaum AS Akin C Wu Y Rottem M Goff JP Beaven MA. Characterization of novel stem cell factor responsive human mast cell lines LAD 1 and 2 established from a patient with mast cell sarcoma/leukemia; activation following aggregation of FcepsilonRI or FcgammaRI. Leuk Res (2003) 27(8):677–82. 10.1016/S0145-2126(02)00343-0 12801524

[22] Ding Y Che D Li C Cao J Wang J Ma P. Quercetin inhibits Mrgprx2-induced pseudo-allergic reaction via PLCgamma-IP3R related Ca(2+) fluctuations. Int Immunopharmacol (2019) 66:185–97. 10.1016/j.intimp.2018.11.025 30471617

[23] Lansu K Karpiak J Liu J Huang XP McCorvy JD Kroeze WK. In silico design of novel probes for the atypical opioid receptor MRGPRX2. Nat Chem Biol (2017) 13(5):529–36. 10.1038/nchembio.2334 PMC5391270 28288109

[24] Nilsson G Blom T Kusche-Gullberg M Kjellen L Butterfield JH Sundstrom C. Phenotypic characterization of the human mast-cell line HMC-1. Scand J Immunol (1994) 39(5):489–98. 10.1111/j.1365-3083.1994.tb03404.x 8191224

[25] Butterfield JH Weiler D Dewald G Gleich GJ. Establishment of an immature mast cell line from a patient with mast cell leukemia. Leuk Res (1988) 12(4):345–55. 10.1016/0145-2126(88)90050-1 3131594

[26] Guhl S Babina M Neou A Zuberbier T Artuc M. Mast cell lines HMC-1 and LAD2 in comparison with mature human skin mast cells–drastically reduced levels of tryptase and chymase in mast cell lines. Exp Dermatol (2010) 19(9):845–7. 10.1111/j.1600-0625.2010.01103.x 20545757

[27] Subramanian H Kashem SW Collington SJ Qu H Lambris JD Ali H. PMX-53 as a dual CD88 antagonist and an agonist for Mas-related gene 2 (MrgX2) in human mast cells. Mol Pharmacol (2011) 31(6):1005–13. 10.1124/mol.111.071472 PMC3102546 21441599

[28] Folkerts J Gaudenzio N Maurer M Hendriks RW Stadhouders R Tam SY. Rapid identification of human mast cell degranulation regulators using functional genomics coupled to high-resolution confocal microscopy. Nat Protoc (2020) 15(3):1285–310. 10.1038/s41596-019-0288-6 PMC7197894 32060492

[29] Hermans MAW Schrijver B van Holten-Neelen C Gerth van Wijk R van Hagen PM van Daele PLA. The JAK1/JAK2- inhibitor ruxolitinib inhibits mast cell degranulation and cytokine release. Clin Exp Allergy (2018) 48(11):1412–20. 10.1111/cea.13217 29939445

[30] Spector I Shochet NR Kashman Y Groweiss A. Latrunculins: novel marine toxins that disrupt microfilament organization in cultured cells. Science (1983) 219(4584):493–5. 10.1126/science.6681676 6681676

[31] Azimi E Reddy VB Shade KC Anthony RM Talbot S Pereira PJS. Dual action of neurokinin-1 antagonists on Mas-related GPCRs. JCI Insight (2016) 1(16):e89362. 10.1172/jci.insight.89362 27734033 PMC5053144

[32] Groot Kormelink T Arkesteijn GJ van de Lest CH Geerts WJ Goerdayal SS Altelaar MA. Mast Cell Degranulation Is Accompanied by the Release of a Selective Subset of Extracellular Vesicles That Contain Mast Cell-Specific Proteases. J Immunol (2016) 197(8):3382–92. 10.4049/jimmunol.1600614 27619994

[33] Babina M Wang Z Artuc M Guhl S Zuberbier T. MRGPRX2 is negatively targeted by SCF and IL-4 to diminish pseudo-allergic stimulation of skin mast cells in culture. Exp Dermatol (2018) 27(11):1298–303. 10.1111/exd.13762 30091263

[34] Colin-York H Li D Korobchevskaya K Chang VT Betzig E Eggeling C. Cytoskeletal actin patterns shape mast cell activation. Commun Biol (2019) 2:93. 10.1038/s42003-019-0322-9 30854485 PMC6405992

[35] Frigeri L Apgar JR. The role of actin microfilaments in the down-regulation of the degranulation response in RBL-2H3 mast cells. J Immunol (1999) 162(4):2243–50. 9973500

[36] Smrz D Bandara G Beaven MA Metcalfe DD Gilfillan AM. Prevention of F-actin assembly switches the response to SCF from chemotaxis to degranulation in human mast cells. Eur J Immunol (2013) 43(7):1873–82. 10.1002/eji.201243214 PMC3798040 23616175

