# Supplementary figures and images for: Human Mast Cell Line HMC1 Expresses Functional Mas-Related G-Protein Coupled Receptor 2
Source: Front Immunol. 2021 Mar 15;12:625284. doi: 10.3389/fimmu.2021.625284 (PMC8006456; doi:10.3389/fimmu.2021.625284)

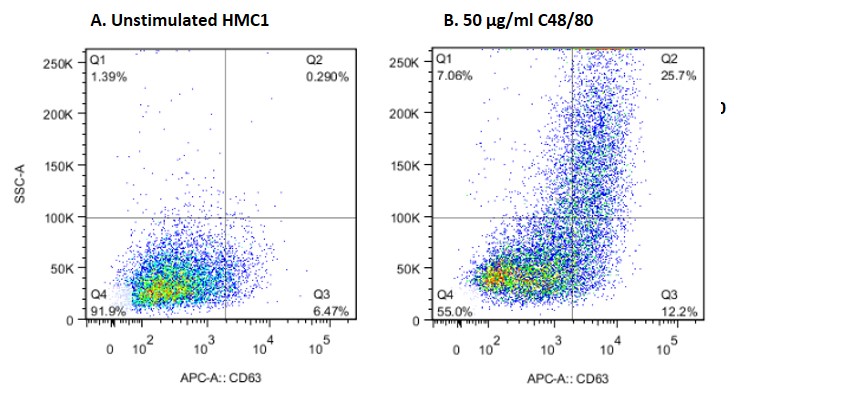

Supplement: Supplementary Figure 1 — Representative example of flowcytometry results for CD63 upregulation upon C48/80 induced degranulation of HMC. Degranulated mast cells are in Q2: they are CD63 positive, and have increase sideward scatter. (A). For the negative control condition, only 0.290% of all cells are degranulated. (B). After stimulation with C48/80, 25.7% of HMC1 are in Q2, thus considered degranulated. [file Image_1.jpeg]

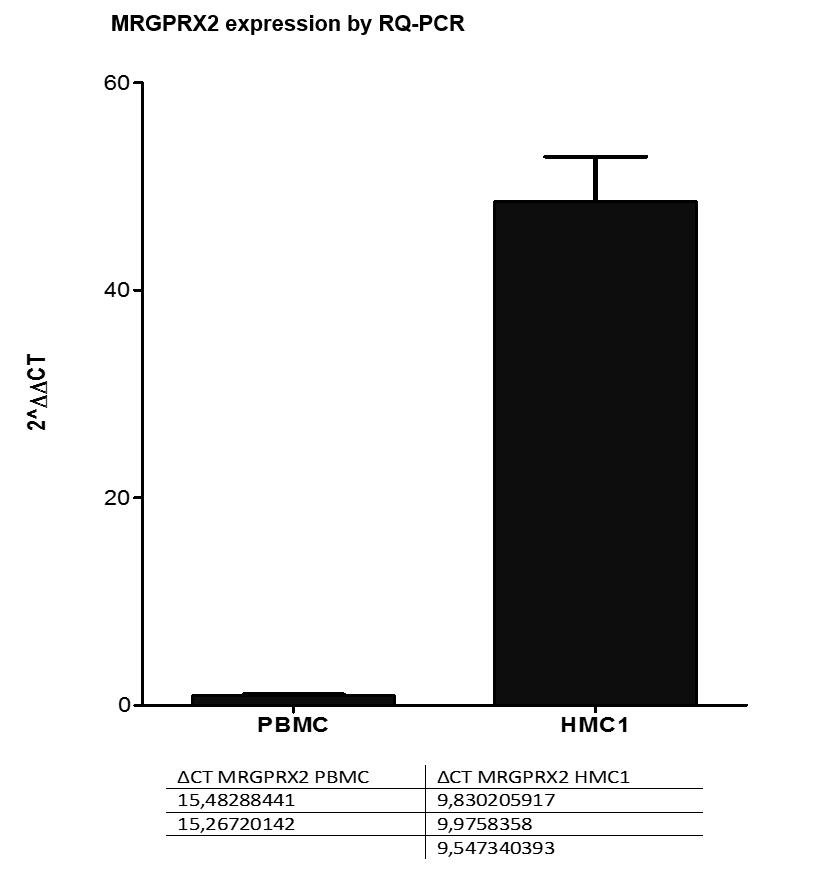

Supplement: Supplementary Figure 2 — Confirmation of MRGPRX2 RNA expression by HMC1. A clear mRNA expression of MRGPRX2 was shown to be present in HMC1 (n=3), but not in PBC (n=2), which were used as negative control. Mean with SEM is shown for CT values. [file Image_2.tif]

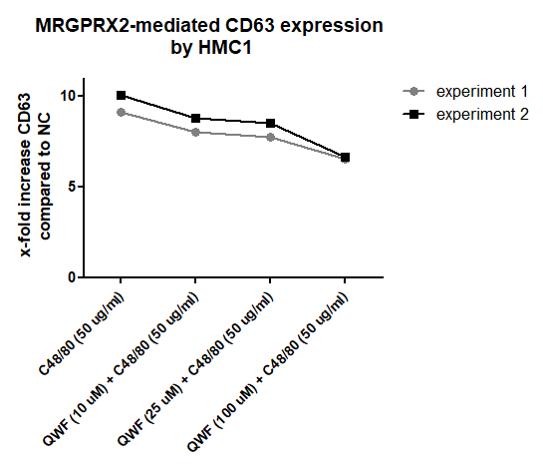

Supplement: Supplementary Figure 3 — QWF dose-dependently inhibits C48/80 induced HMC1 degranulation. A titration of QWF doses was performed to identify the optimally inhibiting dose of QWF. Two representative experiments are shown. HMC1s, pre-incubated with Lat-B, were first incubated with QWF for 10 minutes and subsequently stimulated with C48/80 for 60 minutes. A dose-dependent decrease of QWF on CD63 expression was found. [file Image_3.tif]

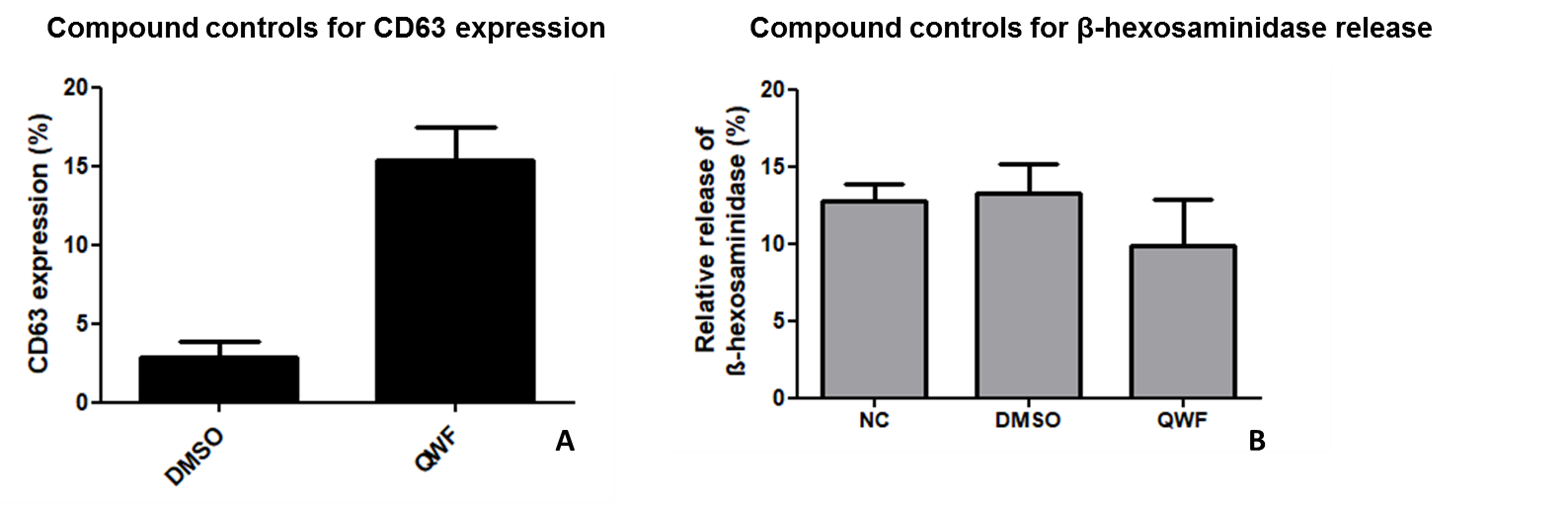

Supplement: Supplementary Figure 4 — Control conditions of the compounds used in this study. The effect of DMSO and QWF alone on the activation of HMC1 was investigated as an extra control. The highest concentrations used in the study were taken: 0.28% DMSO, and 100 µg/ml QWF. (A) Both DMSO and QWF did not induce relevant β-hexosaminidase release (n=3). (B) QWF appeared to induce a nonspecific CD63 upregulation although not statistically significant compared with the diluent control DMSO (n=6). [file Image_4.tif]
