# Supplementary material for: Human Mast Cell Line HMC1 Expresses Functional Mas-Related G-Protein Coupled Receptor 2
Source: Front Immunol. 2021 Mar 15;12:625284. doi: 10.3389/fimmu.2021.625284 (PMC8006456; doi:10.3389/fimmu.2021.625284)
Supplement: Supplementary file 5 [file DataSheet_1.docx]

Supplemental online material belonging to

**Human Mast Cell line 1 (HMC1) expresses functional MRGPRX2.**

###

### Assessing MRGPRX2 expression on RNA level

#### RNA isolation

For the isolation of RNA, cells were lysed in RNA Lysis Solution for total RNA (Sigma-Aldrich) which was supplemented with 1% 2-Mercaptoethanol. GenElute™ Mammalian Total RNA Miniprep kit (Sigma-Aldrich) was used to isolate RNA according to protocol of the company. RNA samples were dried in a SpeedVac concentrator SVC100H (Savant) and taken up in 8.5 μl DEPC treated water (Sigma-Aldrich). RNA concentrations were measured using a NanoDrop 2000 Spectrophotometer (Thermofisher Scientific).

#### Synthesis of cDNA

Of each sample, 2μg RNA was transferred to a tube to which 17 μl of H_2_O (RNase-free) was added. Tubes were incubated for 10 minutes at 70 °C. For each cDNA reaction the following mix was prepared: 4 μl 10X CA buffer (0.2 M Tris pH 8,3, 0.5 M KCl), 8 μl 25 mG MgCl_2_ (Applied Biosystems Life Technologies), 4 μl 100 mM dithiotreitol (DTT, Invitrogen Life Technologies), 2 μl 100 μM random hexamers ((Invitrogen Life Technologies), 1 μl recombinant RNAsin (40 U/μl, Promega), 2 μl Superscript II (200 U/μl. (Invitrogen Life Technologies), 2 μl 20 mM dNTP (GE Healthcare). Of this mix, 23 μl was added to tube containing RNA for the cDNA synthesis reaction. This was mixed and subsequently incubated 45 minutes at 42 °C. Hereafter, the mixture was incubated 3 minutes at 99 °C. cDNA samples were stored -20 °C.

#### rqPCR

Primer probe mixes were ordered for GAPDH (Hs02786624_g1, Thermo Fisher Scientific) and MRGPRX2 (Hs00365019_s1, Thermo Fisher Scientific). Per gene sample combination, a reaction mixture was prepared as follows: 7,5 ul TaqMan Universal Mastermix (Thermofisher Scientific), 0,75 ul selected primer probe mix, 3,75 ul MilliQ, 3 ul cDNA of selected sample. Of the prepared mixture, 10 ul was added to Hard-Shell® 96-well PCR plates (Bio-Rad). For each condition, duplicate reactions were performed. Plates were sealed and subsequently centrifuged for 30 seconds at 500g. rqPCR was run on Taqman 7900HT StepOnePlus. The reaction consisted of incubation for 2 minutes at 50˚C, followed by for 10 minutes at 95˚C, with subsequently by 40 cycles of 15 seconds at 95˚C, ending with 1 minute at 60˚C. Analysis was performed using StepOne^TM^ software (Thermofisher Scientific), with the threshold at 0.06. Relative expression of MRGPRX2 was calculated using GAPDH as the reference housekeeping gene.
